# A steady-state method for computation of myocardial blood volume with the intravascular contrast agent Ablavar

**DOI:** 10.1186/1532-429X-14-S1-P49

**Published:** 2012-02-01

**Authors:** Octavia Biris, Brandon Benefield, Kathleen R Harris, Daniel C Lee

**Affiliations:** 1 Radiology, Northwestern University, Chicago, IL, USA; 2 Biomedical Engineering, Northwestern University, Evanston, IL, USA; 3 Feinberg Cardiovascular Institute, Northwestern University, Chicago, IL, USA; 4 Feinberg School of Medicine, Northwestern University, Chicago, IL, USA

## Summary

Our proof of principle results show the feasibility of quantifying myocardial blood volume (MBV) with the intravascular contrast agent MS-325. For severe stenoses in a canine model, stenosis zone MBV was higher than remote MBV at rest, and lower than remote during vasodilation.

## Background

In contrast echocardiography, absolute MBV in ml/100 g tissue has been shown to correlate with myocardial flow reserve at stress. Furthermore, MBV increases with severity of stenosis at rest, raising the possibility of detecting CAD without a stressor. We show proof of principle that MBV can be quantified by CMR using the intravascular contrast agent MS-325.

## Methods

Protocol: We performed two rest-stress imaging experiments in an instrumented dog with a left circumflex coronary (LCX) artery stenosis created by hydraulic occluder. Severity of stenosis was evaluated by reduction in myocardial blood flow (MBF) measured by microspheres. During both experiments, the animal received 0.006 mmol/kg MS-325 (Ablavar) at rest and again during adenosine stress. T1 mapping was performed before and after contrast administration on a 1.5 T Espree (Siemens, Erlangen, Germany), with the MOLLI pulse sequence (short axis slice 8 mm, FOV 171 x 343 mm2, TR 173 ms, effective TI 100 ms), before and after administration of contrast agent.

### Image Processing

 We have estimated the T1 changes in the myocardium and left ventricle blood pool through fitting of MOLLI signal to the Look-Locker regrowth curve (Signal(t)=M0(1-inf(exp(-t/T1*)) in MATLAB R2009a. MBV was calculated by signal difference maps, according to a slow water exchange model. The final quantitative MBV values were obtained after hematocrit, density and water exchange correction (MBV (ml/100g)= 100*(1/WCF)* 0.95 * (1-HCTventricle)/(1-HCTcapillary)* MBV (%),with a ventricular hematocrit of 0.4, and capillary hematocrit 0.2). We assumed slow water exchange with a correction factor (WCF) of 4, based on previous experiments.

## Results

Measurements of MBV and MBF over the whole myocardium, in the stenosis zone, and in a healthy remote zone are summarized in Table 1. The stenoses caused flow reduction at rest (36% and 25% reduction in MBF), associated with an increase in MBV in the stenosis zone compared to the remote zone (33% and 9.9%). At stress, average MBV increases, but stenosis zone MBV is lower than remote MBV (20.1% and 19.9%). For both experiments, MBV and MBF reserves (MVR and MFR) are in the range of published values.

**Table 1:** MBV by MRI shows expected relationship to MBF by microspheres

Experiment 1					
	Rest		Stress		

	MBV (ml/100g)	% MBV increase (Stenosis-Remote)/Remote	MBV (ml/100g)	% MBV reduction (Stenosis-Remote)/Remote	MVR

Average	4.923		5.771		1.172
Stenosis	6.186	33.0	5.395	20.07	0.872
Remote	4.651		6.75		1.451

Flow from microspheres					

	Rest		Stress		

	MBF (ml/min/g)	% Flow reduction (Stenosis-Remote)/Remote	MBF (ml/min/g)	%Flow reduction (Stenosis-Remote)/Remote	MFR

Stenosis	0.961	35.98	0.714	838.495	0.742
Remote	1.502		8.469		5.638

Experiment 2					

	Rest		Stress		

	MBV (ml/100g)	%MBV increase (Stenosis-Remote)/Remote	MBV (ml/100g)	%MBV reduction (Stenosis-Remote)/Remote	MVR

Average	3.241		4.828		1.490
Stenosis	3.669	9.85	4.335	19.94	1.182
Remote	3.340		5.415		1.833

Flow from microspheres					

	Rest		Stress		

	MBF (ml/g/min)	%Flow reduction (Stenosis-Remote)/Remote	MBF (ml/g/min)	% Flow reduction (Stenosis-Remote)/Remote	MFR

Stenosis	0.687	25.239	0.908	627.226	1.322
Remote	0.918		6.414		8.583

## Conclusions

MBV can be quantified by CMR using the intravascular agent MS-325. Our MBV results mirror the expected vasodilation of microvessels distal to a resting epicardial stenosis, which results in a rest increase of MBV. Under maximal pharmacologic vasodilation, the limitation in epicardial flow results in lower MBV in the stenosis zone relative to the remote zone.

## Funding

None.

**Figure 1:**
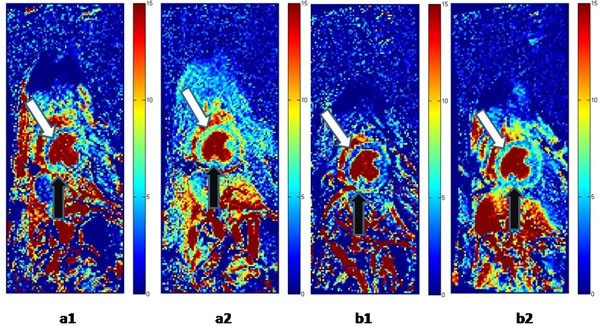
**Myocardial blood volume maps show feasibility of steady state MBV images with adenosine stress.** Quantitative MBV (ml/100g tissue) maps in a canine with LCx stenosis: a1) Severe stenosis at rest causes vasodilation or MBV increase in the LCx bed or inferior myocardium (black arrow) compared to anteroseptal remote regions (white arrow) a2) At stress, inferior area experiences less MBV increase b1) Less severe stenosis shows less marked increase in inferior MBV at rest b2) At stress, inferior MBV increases less than MBV in remote area.

